# Induction of ATP Release, PPIX Transport, and Cholesterol Uptake by Human Red Blood Cells Using a New Family of TSPO Ligands

**DOI:** 10.3390/ijms19103098

**Published:** 2018-10-10

**Authors:** Irene Marginedas-Freixa, Cora L. Alvarez, Martina Moras, Claude Hattab, Guillaume Bouyer, Arnaud Chene, Sophie D. Lefevre, Caroline Le Van Kim, Frederic Bihel, Pablo J. Schwarzbaum, Mariano A. Ostuni

**Affiliations:** 1 UMR-S1134, Integrated Biology of Red Blood Cells, INSERM, Université Paris Diderot, Sorbonne Paris Cité, Université de la Réunion, Université des Antilles, F-75015 Paris, France; neri129@gmail.com (I.M.-F.); martina.moras@inserm.fr (M.M.); chattab@ints.fr (C.H.); arnaud.chene@inserm.fr (A.C.); sophie.lefevre@inserm.fr (S.D.L.); caroline.le-van-kim@inserm.fr (C.L.V.K.); 2 Institut National de la Transfusion Sanguine, Laboratoire d’Excellence GR-Ex, F-75015 Paris, France; 3 Instituto de Química y Fisico-Química Biológicas “Prof. Alejandro C. Paladini”, UBA, CONICET, Facultad de Farmacia y Bioquímica, Junín 956, C1113AAD Buenos Aires, Argentina; alvarezcora@gmail.com (C.L.A.); pschwarzbaum@gmail.com (P.J.S.); 4 Departamento de Biodiversidad y Biología Experimental, Facultad Ciencias Exactas y Naturales, Universidad de Buenos Aires, C1113AAD Buenos Aires, Argentina; 5 UMR 8227 LBI2M, Comparative Erythrocyte’s Physiology, CNRS, Sorbonne Université, Laboratoire d’Excellence GR-Ex, F-29680 Roscoff, France; guillaume.bouyer@sb-roscoff.fr; 6 UMR7200, Laboratoire d’Innovation Thérapeutique, Faculty of Pharmacy, University of Strasbourg, CNRS, F-67400 Illkirch Graffenstaden, France; fbihel@unistra.fr; 7 Departamento de Química Biológica, Facultad de Farmacia y Bioquímica, Universidad de Buenos Aires, C1113AAD Buenos Aires, Argentina

**Keywords:** TSPO, TSPO2, red blood cell, erythrocyte, ATP, ZnPPIX, plasmodium, malaria, VDAC

## Abstract

Two main isoforms of the Translocator Protein (TSPO) have been identified. TSPO1 is ubiquitous and is mainly present at the outer mitochondrial membrane of most eukaryotic cells, whereas, TSPO2 is specific to the erythroid lineage, located at the plasma membrane, the nucleus, and the endoplasmic reticulum. The design of specific tools is necessary to determine the molecular associations and functions of TSPO, which remain controversial nowadays. We recently demonstrated that TSPO2 is involved in a supramolecular complex of the erythrocyte membrane, where micromolar doses of the classical TSPO ligands induce ATP release and zinc protoporphyrin (ZnPPIX) transport. In this work, three newly-designed ligands (NCS1016, NCS1018, and NCS1026) were assessed for their ability to modulate the functions of various erythrocyte’s and compare them to the TSPO classical ligands. The three new ligands were effective in reducing intraerythrocytic *Plasmodium* growth, without compromising erythrocyte survival. While NCS1016 and NCS1018 were the most effective ligands in delaying sorbitol-induced hemolysis, NCS1016 induced the highest uptake of ZnPPIX and NCS1026 was the only ligand inhibiting the cholesterol uptake. Differential effects of ligands are probably due, not only, to ligand features, but also to the dynamic interaction of TSPO with various partners at the cell membrane. Further studies are necessary to fully understand the mechanisms of the TSPO’s complex activation.

## 1. Introduction

Translocator Protein (TSPO) is an 18 kDa ubiquitous transmembrane protein, previously known as the Peripheral Benzodiazepine Receptor (PBR), described as an alternative binding site for benzodiazepines, which is different from the central nervous system’s binding site [1,2].

Two main isoforms of the protein have been identified: (i) TSPO1, which is ubiquitous and mainly present in the outer mitochondrial membrane, and (ii) TSPO2, which is a specific protein of the erythroid lineage, located in the plasma membrane, nucleus, and the endoplasmic reticulum, and is highly expressed in the last developmental stages of the erythrocyte maturation [3,4,5].

Mitochondrial TSPO1 is usually complexed with other protein partners such as the voltage dependent anion channel (VDAC) and the adenine nucleotide transporter (ANT) [6]. Electrophoretic studies have identified protein complexes, ranging from 30 to 200 kDa, involving TSPO, VDAC, ANT and other proteins like the ATPase family AAA Domain-containing protein 3 (ATAD3) [7]. Treatment of those complexes under denaturing conditions fails to completely break the complexed proteins, suggesting the presence of covalent crosslinks between them [8]. Furthermore, we recently demonstrated that TSPO2, VDAC, and ANT are present in the detergent-resistant domains of the erythrocyte membrane [5].

A considerable number of studies have described a broad group of functions in which TSPO is involved, either directly, or in combination with its complex partners; among them, TSPO has been suggested to be involved in cholesterol and porphyrin transport [9,10,11,12]. However, despite the various studies and different TSPO knock-out models which have been developed, the exact function (or functions) of TSPO remain controversial [13,14,15,16].

Nevertheless, almost all the studies describing TSPO functions are based on the actions of TSPO ligands. These ligands constitute a broad range of endogenous and exogenous molecules, [17,18,19] used both to study TSPO involving functions, as well as TSPO tracers for diagnostic imaging [20]. These were originally classified as agonists or antagonists, following thermodynamics constraints [21]. However, this classification fast became obsolete, when it was shown that the same ligand could present activator or inhibitor effects, depending on the analyzed function.

Noteworthy, some ligands are able to bind directly to the protein alone, such as PK 11195 [22,23], but the affinity of others ligands seems to depend on the TSPO involving complex composition [24,25]. In this respect, we recently demonstrated that *P. falciparum* infection, which is known to alter erythrocyte membrane composition, modified the binding of TSPO ligands to the erythrocyte membrane [5].

The discovery of TSPO2 isoform in 2009 [4,26] introduced an extra level of complexity on the study of TSPO functions and binding specificity.

The distinctive activities of the two paralogous proteins may be purely determined by their specialized location, due to the hematopoietic tissue-, erythrocyte-, and organelle-specific expression of TSPO2, but also by the particular complexes’ partners.

We recently characterized two main TSPO functions in the red blood cell (RBC). We identified the mechanism by which TSPO ligands could inhibit the intraerythrocytic development of *P. falciparum* [5], involving zinc protoporphyrin (ZnPPIX) transport and ROS accumulation, and described a novel function of TSPO as an ATP transporter of RBCs [27].

Studies performed with well characterized TSPO ligands PK 11195, Ro5-4864, and SSR-180,575, showed micromolar affinity for erythrocyte membranes expressing TSPO2, as compared with nanomolar affinities of those ligands for TSPO1-expressing membranes.

In the present study, we have compared the effects of the classical ligands (PK 11,195, Ro5-4865, and SSR-180,575), against three imidazoquinazolinone-derivative, newly synthetized TSPO ligands (NCS1016, NCS1018, and NCS1026 [28]), and studied their effects on the main functions identified for erythrocyte TSPO2, which include the release of intracellular ATP [27], uptake of the heme analog ZnPPIX [5], and *P. falciparum* growth [5,29], as well as cholesterol uptake.

These new TSPO ligands have shown higher or lower effect, compared to classical ligands, depending on the analyzed function. While, NCS1018 presented both higher anti-plasmodium activity and ATP release induction, NCS1016 showed significantly higher effect stimulating ZnPPIX uptake and anti-plasmodium activity, and NCS1026 was the only ligand to inhibit cholesterol incorporation into erythrocyte membranes.

The study of the newly synthetized TSPO ligands presented, herein, reveals a path forward on the study and modulation of TSPO functions, in which the fine-tuning of ligand-protein interactions appears as a crucial point in controlling the modulation of the protein’s or protein complex’s function.

## 2. Results

### 2.1. Binding-Affinity of the Translocator Protein (TSPO) Ligands

The affinity of the ligands for the RBC membranes was assessed by typical displacement experiments. RBCs were exposed to 10 nM (^3^H) PK 11195 and increasing concentrations of non-radiolabeled TSPO ligands. All ligands showed low-micromolar affinities for the erythrocyte membranes (Table 1).

These results provided two interesting pieces of information. First, all three new ligands showed better affinity (vs. (3H) PK 11195) for the TSPO2-expressing membranes than the classical ligands Ro5-4864 and SSR-180,575. Second, the IC_50_ values obtained from TSPO2-expressing RBC membranes, as compared to those obtained with TSPO1-enriched rat heart microsomes [28], were one-order lower for both NCS1016 (0.45 vs. 0.058 µM, respectively) and NCS1026 (0.28 vs. 0.01 µM, respectively), supporting the hypothesis that the binding domains for these ligands were significantly different for both isoforms.

### 2.2. P. falciparum Growth Inhibition

We have recently characterized the mechanism by which the TSPO ligands can inhibit a parasite’s growth, a process involving ZnPPIX transport and ROS accumulation [5].

**Figure 1:**
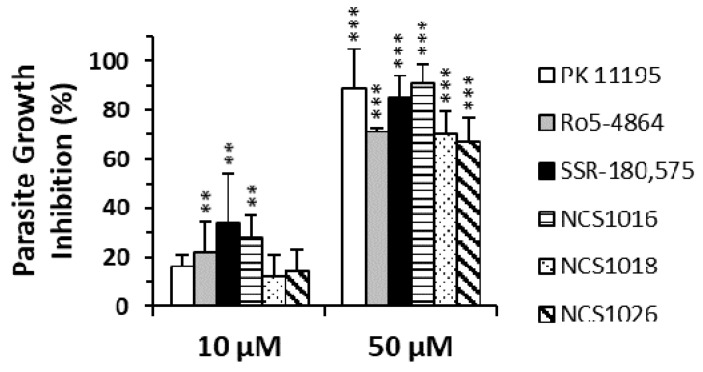
TSPO ligands inhibited the *P. falciparum* growth. Infected RBCs were diluted to 1% parasitemia, and maintained at 1–5%, by dilution, for 48 h. Percentages of growth-inhibition were obtained, after two parasite cycles, by the flow cytometry analysis of parasitemia, and normalized to the control condition (solvent-treated cells). Data are presented as the mean ± SEM; *N* = 4. Differences between the control condition and the treatment were considered significant at ** *p* < 0.01; *** *p* < 001.

The inhibition of parasite’s growth, caused by the newly synthesized TSPO ligands NCS1018, NCS1016, and NCS1026 is shown in Figure 1, and is compared with the results obtained previously with PK 11195, Ro5-4864, and SSR-180,575. The inhibition of parasite-growth, by all three TSPO ligands, were significantly higher at 50 µM than at 10 µM, reaching 80–100% growth inhibition at a concentration of 50 µM. NCS1016, together with SSR-180,575, showed the highest anti-plasmodium activity trend.

Interestingly, the TSPO ligands used, herein, had no detectable effect on hemolysis, further suggesting that their antiparasitic effect did not compromise the integrity of the RBCs membrane (Table 2).

### 2.3. ZnPPIX Uptake and Reactive Oxygen Species (ROS) Accumulation

In our previous work, we identified the way by which the TSPO ligands were able to compromise *P. falciparum* development within the RBC. We showed that the uptake of the fluorescent heme analogue ZnPPIX was enhanced by TSPO ligands, impairing parasite growth, by altering the heme detoxification pathways of the parasite inside the RBC [5].

As mitochondrial TSPO was previously shown to be involved in the transport of porphyrins and heme analogues, we tested whether a similar mechanism could take place with TSPO2 in healthy and infected RBCs. Cells were incubated with ZnPPIX, a fluorescent heme analog, prior to TSPO ligands exposure. In both healthy and infected RBCs, basal ZnPPIX uptake was observed, while enhanced uptake of this compound was induced by the TSPO ligands.

Increased levels of porphyrins, within the cells, had no cytotoxic effect on infected or non-infected RBCs, but induced an increase in the accumulation of reactive oxygen species (ROS), that was significantly enhanced in the presence of the TSPO ligands. While in healthy RBCs, the entry of ZnPPIX could be buffered by the existence of detoxifying glutathione systems, in infected RBCs, the buffering system was not able to compensate for ROS increase, as the glutathione reducing system had already been diminished by the parasite’s entry.

**Figure 2:**
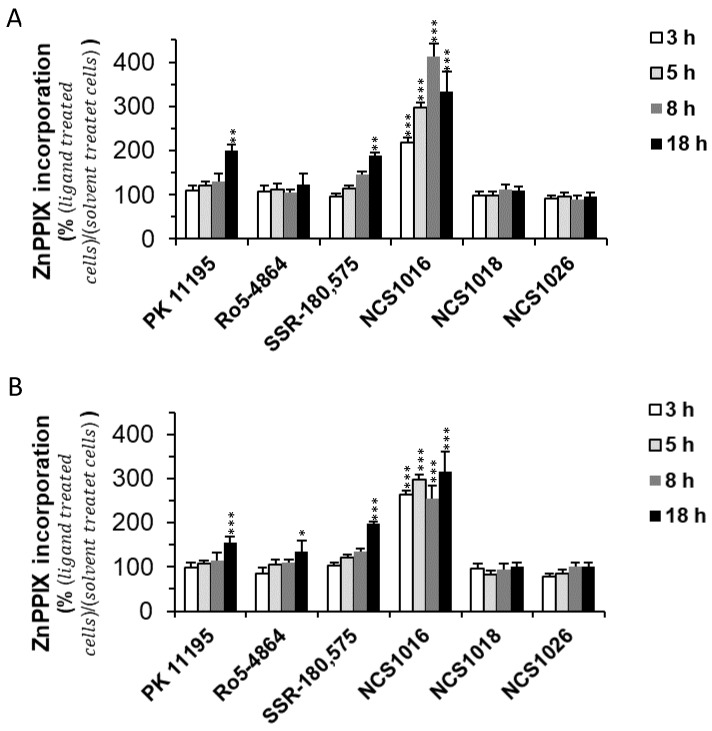
ZnPPIX uptake in healthy and infected RBCs induced by TSPO ligands. Healthy RBC (**A**) and infected RBC (**B**) at 2 to 5% parasitemia were incubated in the presence of 20 µM ZnPPIX and 50 µM TSPO ligands, and the uptake of ZnPPIX was assessed at different time points by flow cytometry. MFI values were normalized to background levels for the control and the ligand-treated conditions. Data are presented as the mean ± SEM; *N* = 7. Statistical analyses were performed for each ligand and the control condition. Differences were considered significant when * *p* < 0.05; ** *p* < 0.01; *** *p* < 0.001.

The new ligands studied, herein, were also tested for their ability to induce an increase in the ZnPPIX uptake (Figure 2). Ligand NCS1016 showed the fastest and highest effect among the TSPO ligands tested. ZnPPIX intracellular levels were already significantly higher than in control cells, after 3 h of incubation, and throughout the whole period, whereas, for the other ligands this increase was not significant until exposure for at least 18 h.

Interestingly, the other two new TSPO ligands, NCS1018, and NCS1026, had no significant effect in inducing an increase in the ZnPPIX uptake, suggesting that TPSO might have other cellular functions besides PPIX uptake.

**Figure 3:**
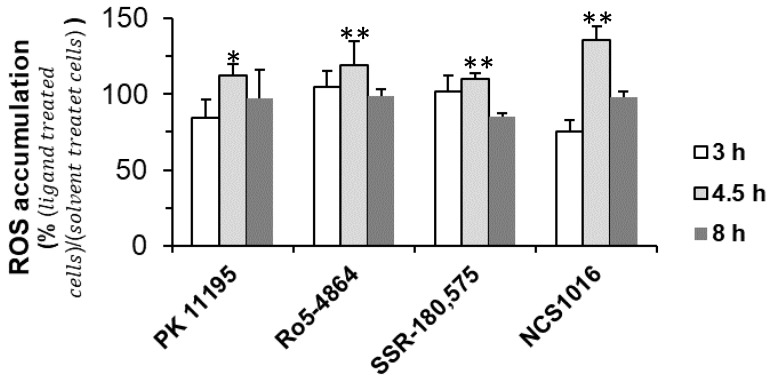
Reactive oxygen species (ROS) induced by TSPO ligands in the presence of ZnPPIX. Infected RBC at 2 to 5% parasitemia were incubated in the presence of 20 µM ZnPPIX and 50 µM TSPO ligands. At several time points samples were washed and incubated with DCFDA and analyzed by flow cytometry. MFI values were normalized to control background levels. No compensation was needed as monostained samples showed no interference between ZnPPIX and the ROS probes. Data are presented as the mean ± SEM; *N* = 8. Statistical analyses were performed for each ligand and control condition. Differences were considered significant when * *p* < 0.05; ** *p* < 0.01.

Accordingly, incubation of the infected RBCs, in the presence of ZnPPIX and the ligand NCS1016, caused an accumulation of ROS, which peaked after 4.5 h of incubation. Once again, the amount of ROS accumulation, induced by ligand NCS1016, was higher than with any of the other previously-tested ligands (Figure 3).

### 2.4. Sorbitol Hemolysis

It was previously proposed that the *P. falciparum* entry into the RBCs, activates a so-called new permeability pathways (NPP), mediating uptake of sorbitol and a wide variety of solutes [30]. Thus, in infected RBCs, addition of sorbitol, in isosmotic conditions, leads to its rapid uptake, together with osmotically-obliged water influx, leading to swelling and hemolysis [31].

We have previously demonstrated that a complex, including TSPO2 and VDAC, was involved in sorbitol transport and that the TSPO ligands could modulate this process by delaying the sorbitol-induced hemolysis, probably by a transport mechanism extruding sorbitol from the cell [5].

**Figure 4:**
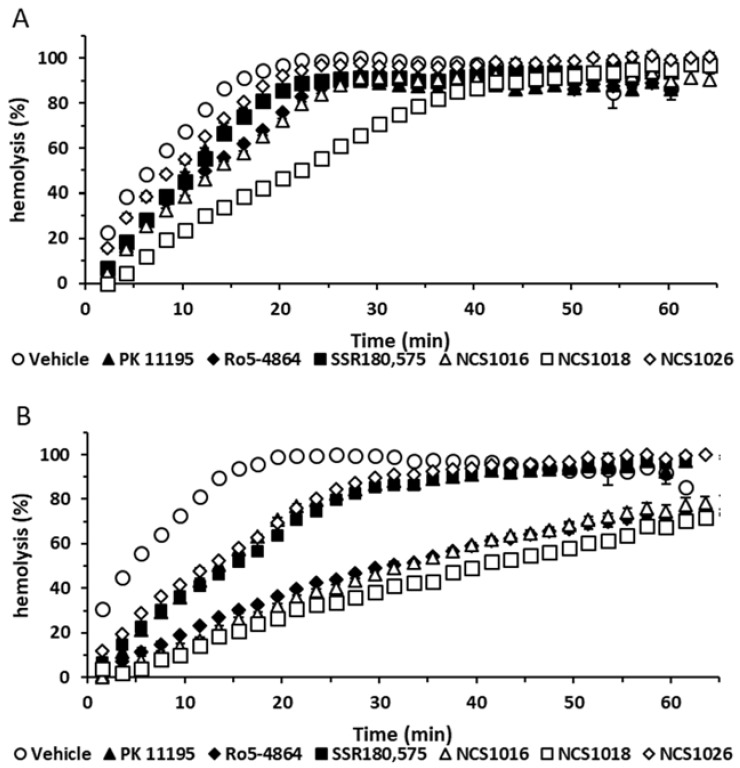
Sorbitol-induced hemolysis is modulated by the TSPO ligands in infected red blood cells. Infected RBCs at 2 to 5 % parasitemia were washed three times in a culture medium, without serum, and resuspended at 50% hematocrit in a media containing 10 μM (**A**) or 50 μM (**B**) TSPO ligands. Hemolysis was quantified at several time-points, by absorption at 540 nm wavelength. Data are presented as mean ± SEM; *N* = 4. Differences between each ligand and control condition (vehicle) were considered significant when *p* < 0.01.

Present results show that the six, tested TSPO ligands were able to delay the sorbitol-induced hemolysis (Figure 4). As shown in Table 3, two qualitatively different responses elicited by ligands could be distinguished. The most effective group of ligands delaying hemolysis kinetics included Ro5-4864, NCS1016, and NCS1018. Differences were more evident at 50 µM, where these compounds showed eight-fold higher t_1/2_ values for Ro5-4864, NCS1016, and NCS1018, and three-fold higher values for SSR-180,575, NCS1026, and PK 11195, than the control samples.

### 2.5. Cholesterol Uptake

One of the TSPO’s most studied function is cholesterol transport [9,10,22]. TSPO1 has been shown to transfer cholesterol across the outer mitochondrial membrane, when stimulated with TSPO ligands. As TSPO2 exhibits both the cholesterol binding domain in the C-terminal domain and the ability to transfer cholesterol [4], we wondered whether cholesterol transport was functional in cell membranes of RBCs, both in healthy and *P. falciparum* infected conditions.

**Figure 5:**
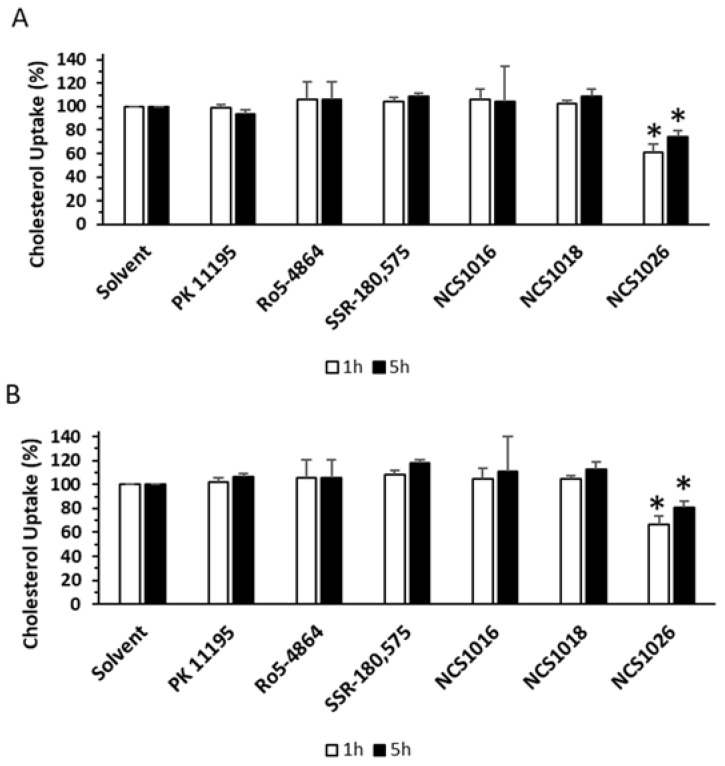
Cholesterol uptake induced by TSPO ligands. Healthy (**A**) or infected red blood cells, at 2 to 5% parasitemia (**B**) were incubated in the presence of TopFluor Cholesterol and 50 µM of TSPO ligands. Cholesterol uptake was assessed at different time points by flow cytometry. MFI values were normalized to vehicle treated cells. Data are presented as mean ± SEM; *N* = 4. Differences between each ligand and control condition (vehicle) were considered significant when * *p* < 0.05.

Infected and healthy RBCs were incubated in the presence of fluorescent cholesterol. The amount of cholesterol inside the cells was assessed by flow cytometry. High amounts of cholesterol were already incorporated in, both, the healthy and the infected RBCs, after 1 h of incubation. Addition of NCS1016, NCS1018, Ro5-4864, and SSR-180,575 did not alter cholesterol uptake in cells. However, NCS1026 inhibited the cholesterol uptake by 40% at 1 h of incubation, while at 5 h, it decreased by 20% (Figure 5).

Interestingly, the efficacy of the NCS1026 correlated with its high-binding affinity to the RBC membranes shown in Table 1.

### 2.6. ATP Release

ATP release from RBCs contributed to the control of the blood flow and tissue-oxygenation [32]. This release can be achieved by the cells lysis, or by a release mechanism mediated by transport proteins or channels. The most well-known protein through which ATP release is achieved, is the pannexin-1which is able to form channels in the RBC membrane that are highly permeable to ATP. However, in recent years several proteins have been postulated to constitute an alternative active pathway for the non-lytic ATP release, and among them, the TSPO partners the VDAC [33].

RBCs play a fundamental role in the tissue oxygen supply, via the controlled release of ATP in the areas of increased oxygen need [34] ATP exit can be achieved by hemolysis or by regulated mechanisms mediated by transporters, channels, or pores [27,32,33,35,36,37]. Among the latter is pannexin-1, a protein thought to arrange in homohexameric pores capable of allowing the transport of ATP across the plasma membrane [38]. However, activation of specific ATP conduits in RBCs appears to depend on the nature of the stimuli under study and the metabolic status of the cell. Accordingly, several proteins have been postulated to constitute alternative pathway for non-lytic ATP release, among them, the TSPO partners the VDAC [33].

In line with the postulated role for the TSPO–VDAC in mediating ATP release from RBCs, we recently provided evidence that the TSPO ligands Ro5-4864, TRO19622, and NCS1018 [27] triggered non-lytic ATP efflux from these cells.

The induced dose-dependent ATP release required VDAC polymerisation and was not affected by the addition of Pannexin-1 blockers. Interestingly, ATP release was blocked by the addition of inhibitors for VDAC and ANT, the latter being another partner of the TSPO2 complex present in RBC membranes.

**Figure 6:**
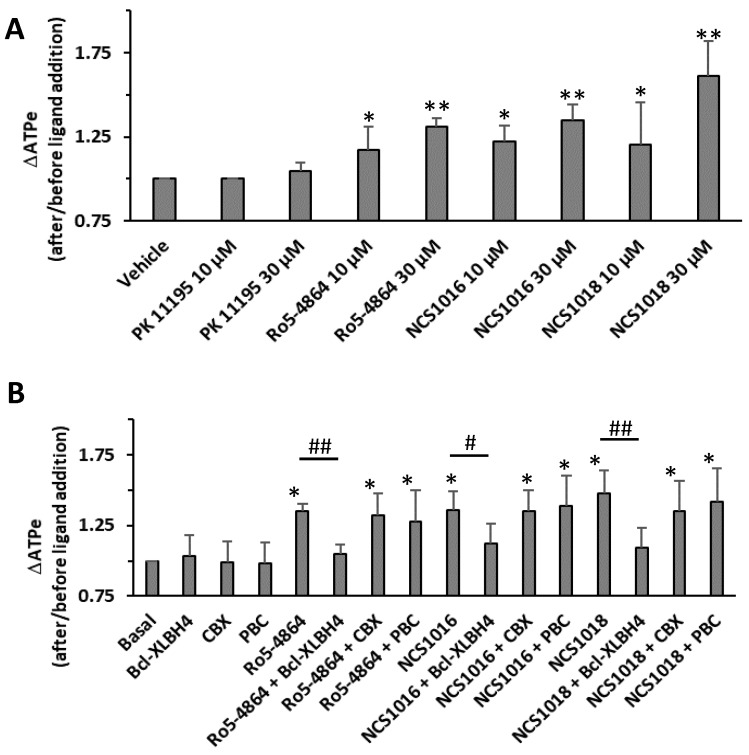
TSPO ligand-induced ATP release. ATP release from human red blood cells was measured at 37 °C, 10% Hematocrit, as described in material and methods. Values are the ratio between extracellular ATP (ATPe) levels after and before the addition of TSPO ligands. Panel (**A**) shows the effect of 10 or 30 µM of PK 11195, Ro5-4864, NCS1016, and NCS1018. Panel (**B**) shows the effect of VDAC inhibitor Bcl-XLBH4 (10 µM), or Pannexin-1 inhibitors carbonexolone (CBX, 10 µM), or probenecid (PBC, 10 µM). Results are expressed as means ± SEM and considered different from basal (vehicle, unstimulated) values when * *p* < 0.05; ** *p* < 0.01 or from those incubated in the presence of Bcl-XLBH4 when ^#^ *p* < 0.05 and ^##^ *p* < 0.01; *N* = 7.

As shown in Figure 6, NCS1018 was previously shown to be the most efficient in inducing ATP release, while the induction potencies of NCS1016 and Ro5-4864 were a bit lower (Figure 6A). Importantly, for NCS1016 and NCS1018, as well as for reference ligand Ro5-4864, activation of ATP release was significantly inhibited by the VDAC blocker Bcl-XL_BH4_, but was not affected by the addition of pannexin-1 inhibitors (Figure 6B).

## 3. Discussion

Historically, the TSPO-related functions were studied using several synthetic or endogenous ligands. Nevertheless, results were sometimes contradictory, depending on the animal or cellular model under study, as well as the ligand nature and ligand concentration. In addition, most studies focused on the TSPO protein alone, so that possible contributions of TSPO partners and the binding properties of ligands to the complexed-TSPO were scarcely studied. This is important as TSPO ligands can display different affinities when interacting directly with TSPO [9,22,23,39], or the interface of TSPO and partner proteins, such as VDAC and ANT [24,25]

The results presented, herein, have been obtained with an atypical, scarcely studied, TSPO-involving model, the complexed-form of TSPO2 [5], where this protein interacts with VDAC and ANT in the membrane of mature RBCs. We used three newly synthesized, as well as three well characterized TSPO ligands to analyze the pharmacological and cellular significance of TSPO, especially when RBCs are infected by *P. falciparum*.

New ligands belonging to the imidazo[1-c]quinazolin-5-one-derived family, were tested. NCS1018 has a furan ring which can function as an H bond acceptor, while NCS1026 and NCS1016 present a benzene or 3-chlorobenzene ring which are essentially hydrophobic. NCS1026 has propyl groups on the amide function, one more carbon than the ethyl groups present on NCS1016 and NCS1018. This is a minor modification but could be sufficient to induce a different effect.

Among the three new ligands, NCS1016 appeared as the most efficient in both inducing a parasite′s death and in increasing the intracellular uptake of ZnPPIX. In accordance with our previous study [5], the increase in the intracellular ZnPPIX caused an accumulation of ROS, reaching a peak after 4.5 h. The amount of ROS accumulation caused by the incubation of *P. falciparum*-infected RBCs with ligand NCS1016 was the highest among all tested ligands. Although ligands NCS1018 and NCS1026 were also highly effective on inducing parasite′s death, none of them was able to modulate ZnPPIX′s incorporation in RBCs. Instead, two other TSPO functions were modulated by those ligands–the ATP release from RBCs could be induced by NCS1018 and NCS1026, with the latter being the only effective ligand in modulating cholesterol incorporation into RBC membranes.

Our results showing that Ro5-4864, as well as ligands NCS1018 and NCS1016 triggered an ATP exit, together with our recent report showing that TSPO forms a multimeric complex with VDAC and ANT [5] agreed well with several lines of evidence, suggesting that multimeric complex units, rather than individual proteins, were acting as transport platforms, allowing the release of ATP from RBCs and other cell types [27,40,41]. In this respect, in *P. falciparum*-infected RBCs, as well as in healthy RBCs exposed to oxidative-stress, activation of ATP-release led to an accumulation of extracellular ATP [30,42], and subsequent autocrine/paracrine activation of purinergic receptors [30]. Subsequent purinergic-dependent intracellular signaling was shown to activate NPP pathways in both infected, as well as oxidative-stressed RBCs. In line with this model, we analyzed the sorbitol-induced hemolysis and found that the most effective ligand in the modulation of NPP was NCS1018. Interestingly, this ligand was unable to modulate ZnPPIX transport but, at the same time, was highly effective for inhibiting the parasite′s growth and for inducing the ATP release in non-parasitized RBCs. These results strongly support the link between sorbitol induced hemolysis and ATP release, and suggest the existence of an ATP regulating-function domain within the TSPO protein/complex, whose modulation led to parasite-death in *P. falciparum*-infected RBCs.

Although TSPO1 ligands have been described as neuroprotective agents, TSPO2 ligands may serve as erythroprotective agents, i.e., in light of the antimalarial effects of TSPO ligands on infected RBCs, and the fact that all malaria symptoms occur during the intra-erythrocyte stage of the disease, improved specific ligands for TSPO, might help to design effective treatment strategies against malaria. In short, we show in this work, that the results obtained with a series of TSPO ligands generated by chemical insertion of a typical moiety of TSPO ligands on the quinazolinone ring of the well-known CBR ligand CGS1367 [28].

The differential efficacy on modulating the functions associated with the TSPO complex, as well as the different affinity for the TSPO complex included in the RBC membrane, suggests that the interaction with the protein or the protein complex was not occurring in the same structural domain.

Moreover, present results suggest that, as for TSPO1, TSPO2 could be involved on different functions, depending on complex composition, and the differential modulation of specific ligands. The identification of the specific ligand-interacting structural domains, within the protein or the protein complex, would help in identifying the crucial domain involved in the function’s modulation.

As we have previously reported, both classical as well as the new synthetic TSPO ligands need micromolar concentrations to modulate RBC functions. Such values contradict the well-characterized TSPO ligand affinities that are usually found in the nanomolar range for the TSPO1 isoform [43,44,45]. This apparent contradiction could be explained by the fact that TSPO2 seemed to be the main isoform at the RBC membrane [5], and this isoform presented a high nanomolar affinity for cholesterol but a dramatically diminished affinity for PK 11195 [4].

All TSPO ligands tested in the present study (PK 11195, Ro5-4864, SSR-180,575, and NCSs) had, as a common feature, a strong lipophilic character. This was consistent with the structure of the TSPO/PK 11195 complex where the PK 11195 was located in a pocket characterized by hydrophobic amino acids [23,46]. The hydrophobic pockets were relatively non-selective, unlike the acceptor/donor H-bonds or electrostatic interactions, and it is possible that the ligands of the TSPO penetrated in a different manner, resulting in different conformational changes responsible for the various activities observed. However, we could not completely exclude the possibility of a TSPO-independent effect, by interaction with other proteins [47], or by unspecific interaction with the lipid bilayer [17,48].

The present study also demonstrated the necessity and efficacy of a new field of research, the development of new TSPO ligands, highly specific for each TSPO isoform, to allow a more specific modulation of protein function and thus help obtain a better understanding of structure-function relationships, and the cellular role of TSPO and its protein partners.

## 4. Materials and Methods 

### 4.1. Red Blood Cell Samples

Human blood was obtained by venipuncture from healthy volunteers the day each study was performed. Immediately after blood collection, plasma, platelets, and leukocytes were removed by centrifugation (900× *g* at 20 °C for 3 min). The supernatant and buffy coat were removed and discarded. The RBCs were resuspended and washed three times in modified Krebs buffer (MKB) (137 mM NaCl, 2.7 mM KCl, 1.5 mM KH_2_PO_4_, 4.72 mM Na_2_HPO_4_, 1.32 mM CaCl_2_, 1.91 mM MgSO_4_, and 5 mM glucose, pH 7.4, 300 mOsM). The packed-RBCs were resuspended in MKB supplemented with 0.5% bovine serum albumin (BSA), to the corresponding final hematocrit. Experiments in this study were performed with RBCs purified from fresh blood. This study was conducted according to institutional ethical guidelines of the National Institute for Blood Transfusion (INTS, Paris, France). Identification code: 665850, date: 16 January 2016. All procedures were carried out in accordance with the Declaration of Helsinki. Written informed consent was given by the donors.

### 4.2. Chemicals

**Figure 7:**
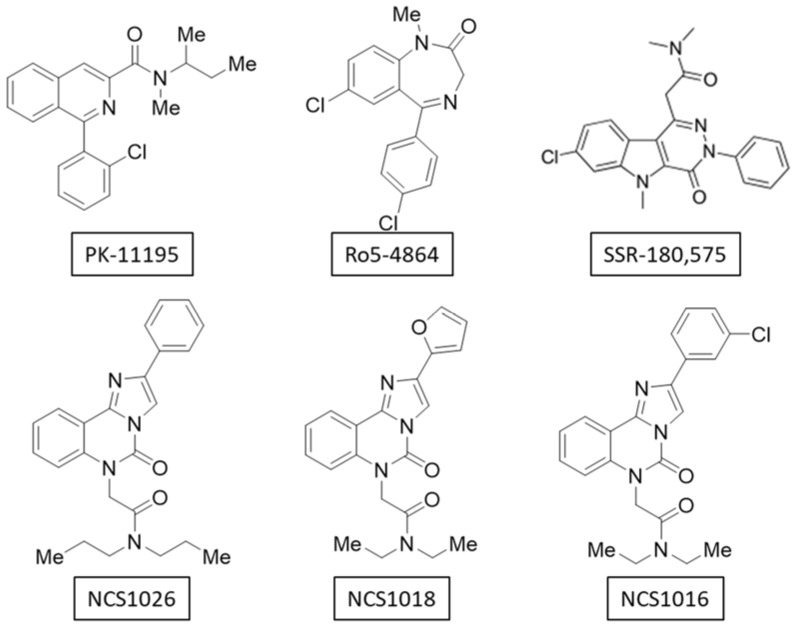
Chemical structure of the TSPO ligands. The six TSPO ligands used in this study belonged to the isoquinolines (PK 11195), benzodiazepines (Ro5-4864), pyridazinoindoles (SSR-180,575), and imidazoquinazolinone-derivative (NCS1016, NCS1018 and NCS1026) families.

Otherwise indicated, all chemicals and solvents used were purchased from Sigma–Aldrich (Marne la Coquette, France). TSPO ligands (Figure 7) *N*,*N*-Diethyl-2-(2-(furan-2-yl)-5-oxomidazo[1-c]quinazolin-6(5H)-yl)acetamide (NCS1018), *N*,*N*-dipropyl-2-(5-oxo-2-phenylimidazo[1-c]quinazolin-6(5H)-yl)acetamide (NCS1026), and *N*,*N*-diethyl-2-(2-(furan-2-yl)-5-oxoimidazo[1-c]quinazolin-6(5H)-yl)acetamide (NCS1016), which belonged to the imidazo[1-c]quinazolin-5-one family, were synthesized following the previously-described protocol [28]. SSR-180,575 was synthetized as previously described [49]. PK 11195 and Ro5-4864 were purchased from Sigma–Aldrich.

### 4.3. Radioligand Binding Assays

RBCs were washed twice in phosphate buffer saline (PBS) (pH 7.4). Samples were incubated in a final incubation volume of 0.3 mL, in the presence of (^3^H) PK 11195 (Perkin Elmer, Courtaboeuf, France; 83.5 Ci/mmol) and increasing concentrations of unlabeled TSPO ligands, at room temperature (RT). After 30 min incubation, assays were stopped by filtration through Whatman GF/C filters. Radioactivity trapped on the filters was determined by liquid scintillation counting (Tri-Carb 2800TR, Perkin-Elmer, Courtaboeuf, France).

### 4.4. ATPe Measurements

Extracellular ATP (ATPe) concentration was measured by quantitative luminometry using firefly luciferase, which catalyzes the oxidation of luciferin in the presence of ATP to produce light as previously described [27,36,50]. The ATPe concentration was determined at discrete times (offline). Changes in [ATPe] over time were used to estimate ATP release from RBCs.

10 µL of RBCs at 10% hematocrit were incubated for 10 min, at 37 °C, in the presence of the ligand, or of an equal vehicle volume, with 30 µL of MKB medium. At discrete times, samples were centrifuged at 450× *g* for 3 min, at RT. Paired samples were taken to assess ATPe and hemolysis. The supernatants were stored at −20 °C, prior to the analysis. Then, 4.5 µL of the supernatant samples were added to 45 µL of the luminometry mix (MKB medium containing 0.01 µM luciferase, 0.2 mM luciferin, and 0.1 mg/mL of Coenzyme A) and placed in the assay chamber of the luminometer to determine the light intensity. At the end of the incubations, the time course of light emission was transformed into the ATPe concentration versus time using a calibration curve, with ATP stock solutions ranging from 1 to 50 µM. The results were expressed as ΔATPe (i.e., the ratio of [ATPe] before and after the addition of a given stimulus).

The increase in [ATPe] due to regulated ATP release was calculated by taking the experimentally-measured [ATPe] and subtracting the estimated [ATPe] due to hemolysis. Except where otherwise stated, the results of ATP release experiments reflected the ATP release from non-lytic origin.

### 4.5. Measurement of Hemolysis and Estimation of Lytic ATPe

Hemolysis leads to hemoglobin (Hb) release into the extracellular milieu, where the Hb content can be quantified by monitoring light absorption, at 405 nm. Using this procedure, we previously observed that the amount of Hb was proportional to the amount of lysed RBCs [50]. The Hb content of the samples was determined as described above. Then, the amount of lytic ATP contributing to [ATPe] was estimated from the total amount of hemolysis cells (calculated as described above) and the intracellular ATP content of the RBCs, which under the experimental conditions was 148 pmol ATP per 10^6^ RBCs, as previously reported by our group [50].

### 4.6. P. falciparum Culture with TSPO Ligands

*P. falciparum* strain FCR3 culture was performed in RPMI 1640 medium containing 10% Albumax, at 5% hematocrit, in a 5% O_2_, 3% CO_2_ atmosphere. Cultures were sorbitol-synchronized for one parasite life-cycle, before experiments were performed. Parasite’s viability assays were started at 1% parasitemia in trophozoite stage; parasitemia was diluted five-fold (to 1% in control condition) after 48 h, so as to allow the parasite’s exponential growth throughout the assay. TSPO ligands were added every 48 h (a complete parasite’s life-cycle) throughout the assay together with the renewal of the RPMI medium, from ethanol or dimethyl sulfoxide (DMSO) stock solutions. Control conditions were performed in the presence of the corresponding solvent. Parasitemia was determined after 48 h and 96 h (after one or two complete parasite’s cycles, respectively) by flow cytometry (FACS), as previously described [5].

### 4.7. Sorbitol Hemolysis Assays

Sorbitol hemolysis assays were carried as previously described [5] using hemoglobin release to estimate lysis time. Monoexponential functions were fitted to the data from the sorbitol-induced hemolysis (%) (Figure 4), at different times of exposure to the ligands. Half time values (t_1/2_) for hemolysis were then calculated as t_1/2_= ln (2)/k, where k is the rate coefficient of the exponential curve.

### 4.8. ZnPPIX Uptake

*P. falciparum* cultures were incubated at 2–5% parasitemia (synchronized cultures, ring and trophozoite stages) in 200 µL of RPMI 10% Albumax containing 20 µM ZnPPIX. TSPO ligands were added at 10 µM and 50 µM. Cultures were kept at 37 °C, in the dark, for 3 to 18 h and further analyzed by FACS.

### 4.9. ROS Accumulation

ROS accumulation was measured using the cell permeant ROS probe 2′,7′-dichlorofluorescein diacetate (DCFDA). Cultures pre-incubated with 20 µM ZnPPIX were washed with PBS and incubated for 30 min in 50 µM DCFDA in PBS (pH 7.2), at 0.5% hematocrit, and kept at 37 °C in the dark. After washing, DCFDA signaling was assessed by the FACS (see details below). Infected-RBCs were differentially stained with the nucleic acid dye TOPRO-3.

### 4.10. Cholesterol Uptake

Cholesterol uptake was measured using TopFluor Cholesterol and 50 µM of TSPO ligands. Cholesterol uptake was assessed at different time points by flow cytometry. Mean Fluorescent Intensity (MFI) values were normalized to vehicle treated cells.

### 4.11. Fluorescence Measurements by Flow Cytometry

Fluorescence measurements in all samples of the *P. falciparum*’s viability, ZnPPIX uptake, cholesterol uptake, and ROS accumulation assays, were performed with a FACSCanto (BD Biosciences, Rungis, France) and further analyzed with Flowjo-3 software. Infected-RBCs were distinguished from non-infected ones contained in the same culture by TOPRO-3 signaling (1/2, 500), with the negative level established with the same non-infected RBCs used for parasite culture. ZnPPIX (Ex.: 488 nm, Em.: 585/42 nm), TopFluor Cholesterol (Ex.: 495 nm, Em.: 507 nm) and DCFDA (Ex.: 488 nm, Em.: 530/30 nm) levels were measured in both the infected and non-infected RBC populations, results were normalized to the corresponding solvent for each ligand.

### 4.12. Statistical Analysis

In each graph, the data represent the mean ± standard error of the mean (SEM), unless otherwise noted. If indicated, statistical significance was calculated using a one-way analysis of variance, followed by Dunnett’s multiple comparison test, with the GraphPad Prism software (Graphpad Software, La Jolla, CA, USA). Differences were considered significant when *p* < 0.05.

## Figures and Tables

**Table 1:** Translocator Protein (TSPO) ligands’ affinity for RBC membranes.

Ligand	Ro5-4864	SSR-180,575	NCS1016	NCS1018	NCS1026
IC_50_ (µM)	1.5 ± 0.6	1.7 ± 0.5	0.45 ± 0.05	0.59 ± 0.07	0.28 ± 0.06

RBCs were incubated in the presence of 10 nM (^3^H) PK 11195 and increasing concentrations of the non–radiolabeled TSPO ligands Ro5-4864, SSR-180,575, NCS1016, NCS1018, and NCS1026. IC_50_ values were calculated from the displacement curves using the following equation: Y = 100 × (S)^h^/(IC_50_h × (S)^h^), where Y is the percentage of bound [^3^H] PK 11195, S is the unlabeled ligand concentration, and h is the Hill coefficient (1 ± 0.2). Data are presented as the mean ± SEM. *N* = 3.

**Table 2:** Hemolysis induced by TSPO ligands treatment.

Ligand	Vehicle	PK 11195	Ro5-4864	SSR-180,575	NCS1016	NCS1018	NCS1026
**%**	0.64 ± 0.02	0.60 ± 0.06	0.68 ± 0.16	0.70 ± 0.08	0.65 ± 0.09	0.72 ± 0.06	0.52 ± 0.06

Hemolysis was calculated from hemoglobin (Hb) released into the extracellular milieu, quantified by monitoring light absorption at 405 nm. Data are presented as mean ± SEM; *N* = 4.

**Table 3:** Quantification of sorbitol-induced hemolysis t_1/2_ values in the presence of different TSPO ligands.

Ligand	10 µM	50 µM
Vehicle	5.24 ± 0.34	4.04 ± 0.22
PK 11195	7.82 ± 0.72 *	11.88 ± 0.68 *
Ro5-4864	9.76 ± 0.84 *	29.49 ± 0.45 **
SSR-180,575	9.16 ± 0.64 *	12.59 ± 0.62 **
NCS1016	11.67 ± 0.90 *	30.23 ± 1.71 **
NCS1018	17.87 ± 2.57 **	37.15 ± 3.42 **
NCS1026	11.67 ± 0.90 *	10.74 ± 0.30 *

Half-time values (t_1/2_) for sorbitol-induced hemolysis in the presence of different TSPO ligands were calculated by fitting the data following the equation *t*_1/2_ = ln(2)/*k*, where *k* is the rate coefficient of the exponential curve. * *p* < 0.05; ** *p* < 0.01; *N* = 3.

## Abbreviations

ANT	Adenine nucleotide transporter
ATAD3	ATPase family AAA Domain-containing protein 3
ATP	Adenosine Tri Phosphate
ATPe	Extracellular ATP
CBX	Carbonexolone
DCFDA	2′,7′-dichlorofluorescein diacetate
MFI	Mean fluorescence intensity
NPP	New permeability pathway
PBC	Probenecid
PBR	Peripheral benzodiazepine receptor
PBS	Phosphate buffer saline
RBC	Red blood cell
ROS	Reactive oxygen species
RT	Room temperature
TSPO	Translocator protein
VDAC	Voltage dependent anion channel
ZnPPIX	Zinc protoporphyrin
